# Rational Engineering of a Flavoprotein Oxidase for Improved Direct Oxidation of Alcohols to Carboxylic Acids

**DOI:** 10.3390/molecules22122205

**Published:** 2017-12-12

**Authors:** Mathias Pickl, Christoph K. Winkler, Silvia M. Glueck, Marco W. Fraaije, Kurt Faber

**Affiliations:** 1 Department of Chemistry, University of Graz, Heinrichstrasse 28, A-8010 Graz, Austria; Mathias.Pickl@edu.uni-graz.at; 2 Austrian Centre of Industrial Biotechnology, ACIB GmbH c/o Department of Chemistry, University of Graz, Heinrichstrasse 28, A-8010 Graz, Austria; winkler_christoph@hotmail.com (C.K.W.); Si.Glueck@uni-graz.at (S.M.G.); 3 Molecular Enzymology Group, Groningen Biomolecular Sciences and Biotechnology Institute, University of Groningen, Nijenborgh 4, 9747 AG Groningen, The Netherlands; m.w.fraaije@rug.nl

**Keywords:** biocatalysis, alcohol oxidation, aldehyde oxidation, flavoprotein oxidase, protein design

## Abstract

The oxidation of alcohols to the corresponding carbonyl or carboxyl compounds represents a convenient strategy for the selective introduction of electrophilic carbon centres into carbohydrate-based starting materials. The O_2_-dependent oxidation of *prim*-alcohols by flavin-containing alcohol oxidases often yields mixtures of aldehyde and carboxylic acid, which is due to “over-oxidation” of the aldehyde hydrate intermediate. In order to directly convert alcohols into carboxylic acids, rational engineering of 5-(hydroxymethyl)furfural oxidase was performed. In an attempt to improve the binding of the aldehyde hydrate in the active site to boost aldehyde-oxidase activity, two active-site residues were exchanged for hydrogen-bond-donating and -accepting amino acids. Enhanced over-oxidation was demonstrated and Michaelis–Menten kinetics were performed to corroborate these findings.

## 1. Introduction

**Figure 1:**
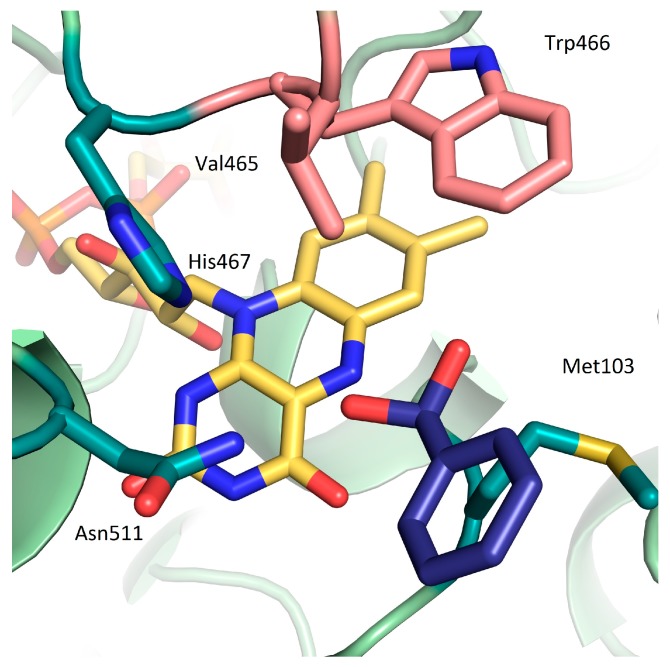
Wild-type HMFO active site (PDB 4UDP): FAD (yellow), residues surrounding flavin-N5 are shown in green (Met103, Asn511, His467), residues selected for site-directed mutagenesis are highlighted in pink (Val465, Trp466), and benzaldehyde hydrate (*gem*-diol of **1b**) is shown in blue.

Oxidases are prominent biocatalysts due to their ability to utilize molecular oxygen as oxidant [1,2]. In contrast to alcohol dehydrogenases, they do not suffer from an equilibrium problem, as the utilization of O_2_ as electron acceptor leads to a practically irreversible reaction [1,3]. Recently, 5-(hydroxymethyl)furfural oxidase from *Methylovorus* sp. MP688 (HMFO, EC: 1.1.3.37), a well expressing, stable flavoprotein, that is active on a variety of benzylic or allylic *prim*-alcohols was described [4], which was termed for its ability to oxidize 5-(hydroxymethyl)furfural [5]. The latter can be obtained from hexoses on a large scale, and its oxidation to 2,5-furandicarboxylic acid furnishes a bio-based monomer for PEF as a substitute for petroleum-derived PET [6]. Furthermore, the oxidation activity of HMFO on *prim*-thiols has been described [7]. Crystal structures of HMFO containing oxidized and reduced flavin are available (Figure 1, PDB: 4UDP and 4UDQ), which makes it a perfect template for rational protein redesign [8]. Analogous to other structurally related flavoprotein oxidases, the reaction proceeds through proton abstraction from OH through a histidine residue (His467), with concomitant hydride transfer to the N5 of the FAD cofactor. Re-oxidation of FADH by O_2_ leads to the formation of hydrogen peroxide and regenerates FAD. In order to avoid detrimental effects of the formed hydrogen peroxide and to regenerate molecular oxygen, catalase was added to the standard reaction setup in this study.

**Scheme 1:**
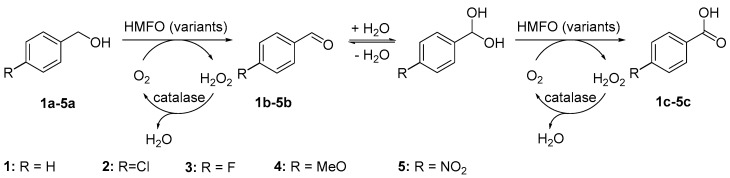
Two-step oxidation of benzylic alcohols to carboxylic acids catalyzed by HMFO variants.

When *prim*-alcohols are used as substrates, the oxidation reaction does not necessarily stop at the aldehyde stage, but may proceed to the corresponding carboxylic acid. This takes place via spontaneous (non-enzymatic) formation of the aldehyde (*gem*-diol) hydrate. In contrast to the aldehyde, this hydrated species is accepted as a substrate by HMFO and furnishes the carboxylic acid via “over-oxidation” (Scheme 1). Similar activities are known for several flavin-dependent alcohol oxidases [9,10,11]. In contrast, Cu-containing galactose oxidase is unable to oxidize aldehydes and/or the corresponding hydrates [12,13,14]. For such enzymes, an additional aldehyde oxidase is required to produce carboxylic acids from alcohols [15].

Although several flavin-dependent alcohol oxidases are capable of oxidizing an alcohol to the corresponding carboxylic acid, they show astonishing differences: some alcohol oxidases readily oxidize the aldehyde to the carboxylic acid [16,17], while others (e.g., aryl alcohol oxidase) render the carboxylic acid only in minor amounts [10]. Furthermore, the equilibrium and rate of (non-enzymatic) aldehyde hydration strongly depend on the electronic properties of the substrate and the solvent system [1]. Overall, HMFO oxidizes aldehydes rather slowly. With the aim to develop an oxidase for the direct oxidation of alcohols to the corresponding carboxylic acids via the aldehyde stage, we engineered HMFO oxidase to enhance its catalytic performance for the second aldehyde oxidation step.

## 2. Results and Discussion

Our strategy to improve the oxidation of the aldehyde hydrate via protein engineering focuses on the introduction of hydrogen-bond-donating or -accepting amino acids that support stabilization of the *gem*-diol in the active site, bearing in mind that an additional H-bond adds ~2 kcal·mol^−1^ of binding energy [18]. Similar patterns are found in aldehyde dehydrogenases, where a polar amino acid active site residue promotes aldehyde hydration and hence favors carboxylic acid formation [19]. Potential active-site residues for mutation were identified by considering the orientation and distance of their side chain to the substrate's hydroxy-groups. Five amino acids in the vicinity of FAD are highlighted in Figure 1.

From those, two residues interacting with the (hydrated) aldehyde moiety of the substrate via hydrogen bonding by positioning of the benzylic hydrogen atom in close proximity of N5 were selected (for docking, see SI): Val465, positioned directly on top of N5 of FAD and Trp466, performing π-π stacking with the benzylic moiety of the isoalloxazine ring of FAD (Figure 1) [8,20].

**Figure 2:**
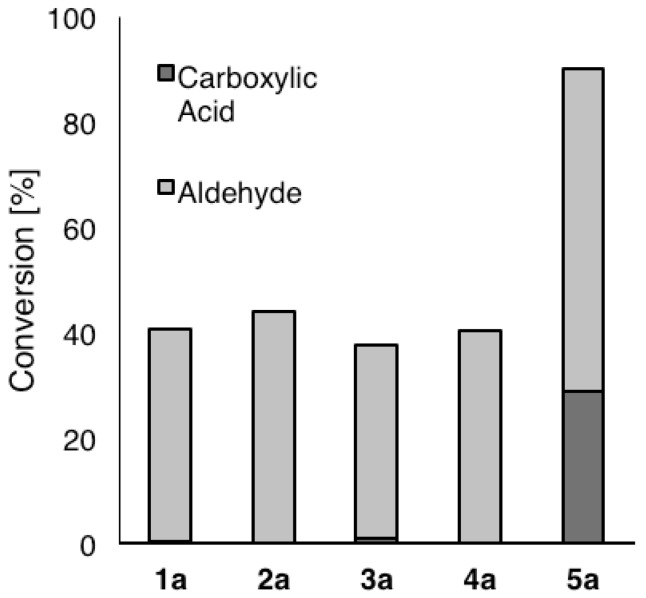
Oxidation of benzylic alcohols **1a**–**5a** (25 mM) by HMFO (wild type, 0.7 µM) in sodium phosphate buffer (100 mM, pH 7.0) at 30 °C with atmospheric oxygen in the presence of catalase after 24 h.

To eliminate potential sources of errors arising from different protein expression levels and background reactions only purified enzymes were tested [21,22,23]. In an initial experiment, the performance of wild-type HMFO in the oxidation of benzylic alcohols was evaluated with substrates bearing electron-withdrawing and -donating groups in the *p*-position (**1a**–**5a**) after 24 h (Figure 2). All substrates were readily oxidized to the corresponding aldehydes **1b**–**5b**. To our delight, the highly activated *p*-nitrobenzyl alcohol **5a** was over-oxidized to carboxylic acid **5c** with 29% conversion, while substrates **1a**–**4a** showed only traces of carboxylic acid formation (<1%). This can be explained by the strong electron-withdrawing *p*-nitro-group of **5a**, which leads to ~15% hydrated *gem*-diol in equilibrium [24]. According to the literature, the ratio between the aldehyde and its (*gem*-diol) hydrate remains constant during a similar enzymatic redox reaction [24,25], which is experimentally supported by the fact that identical endpoint results were obtained for reactions using pre-incubated aldehyde (see SI). This means that the rate of aldehyde hydration is not rate limiting, but the position of the equilibrium.

**Figure 3:**
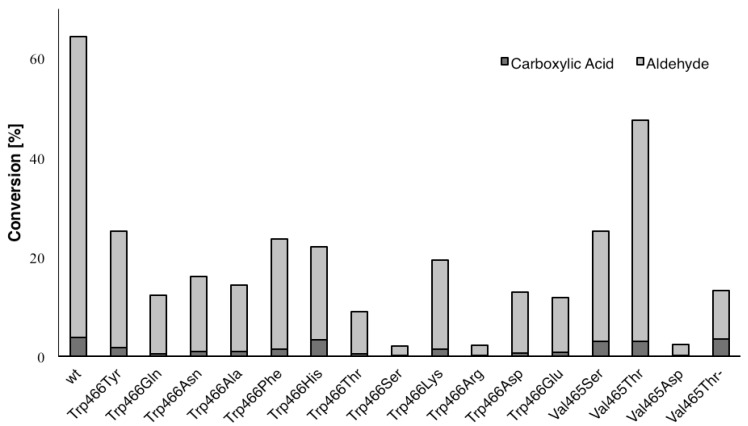
Oxidation of 4-nitrobenzyl alcohol (**5a**) (25 mM) to 4-nitrobenzaldehyde (**5b**) and 4-nitrobenzoic acid (**5c**) by HMFO variants (0.7 µM) in sodium phosphate buffer (100 mM, pH 7.0) at 30 °C with atmospheric oxygen in presence of catalase after 2 h (HPLC).

In order to enhance binding of the aldehyde hydrate, Trp466 and Val465 in wild-type HFMO (Figure 1, purple) were systematically exchanged to amino acids that may serve as H-bond donors or -acceptors, i.e., tyrosine, glutamine, asparagine, histidine, threonine, serine, lysine, arginine, aspartate, and glutamate. In order to differentiate H-bonding from steric effects, also apolar amino acids alanine and phenylalanine were included. Since wild-type HMFO already showed measurable formation of the carboxylic acid after 2 h (**5a**, 4%), this time point was chosen as a reference point for the comparison of variants, because product inhibition and problems of enzyme instability are negligable. With non-activated substrates **1a**–**4a**, all variants gave only traces of over-oxidized product. However, using **5a** as substrate, encouraging results were obtained (Figure 3). Although activities dropped in general, several mutants bearing polar residues in place of Val465 and Trp466 showed enhanced chemoselectivity for carboxylic acid formation relative to wild type HMFO (Table 1).

Most variants showed a similar chemo-selectivity for carboxylic acid/aldehyde formation of 1:12 to 1:15 as the wild type. Although Trp466Ser, Trp466Arg, and Val465Asp variants showed enhanced over-oxidation, their overall activity was disappointing (<5%). In contrast, Trp466His and Val465Ser mutants showed 2–3-fold enhanced acid formation by maintaining reasonable overall activities (~25%).

In an attempt to combine good activity with enhanced chemoselectivity, a double variant was constructed by combining Val465Thr (most active) with Trp466His (highest chemoselectivity) (Figure 3). By this way, the ratio of aldehyde-formation/overoxidation could be further pushed to a 5-fold improvement of the chemoselectivity at the expense of some loss of activity.

The potency of the exchange of Trp466 to His may be explained by two effects: The introduction of a hydrogen bond interaction with the substrate while maintaining π-π interaction with FAD for re-oxidation (similar to Trp at this position in the wild type) [26]. The positive effect of Ser in position 465 may be attributed to maintaining Trp466 in place for efficient re-oxidation [20] combined with favorable H-bonding of the aldehyde hydrate.

For further characterization of the activities of enzyme variants in the individual oxidation steps, Michaelis–Menten kinetics were determined. Hence, alcohol (**5a**) and aldehyde (**5b**) were tested with the wild-type enzyme and the most active variants Val465Thr and Trp466His (Table 2). The catalytic efficiency of the wild type enzyme was found to be high for **5a** (*k*_cat_/*K*_M_ 28 s^−1^·mM^−1^) and modest for **5b** (~1 s^−1^·mM^−1^), accounting for the high chemoselectivity (28-fold) between both oxidation steps. Since the *K*_M_ for alcohol and aldehyde (hydrate) remained about the same (0.26 versus 0.31 mM), chemoselectivity was (almost entirely) caused by differences in *k*_cat_ (7.7 vs. 0.3 s^−1^). The doubled catalytic efficiency of Val465Thr for **5a** was caused by a 10-fold lower *K*_M_, which over-compensates a ~4-fold reduction in *k*_cat_ compared to the wild type. The Trp466His variant showed a three orders of magnitude lower catalytic efficiency, which is due to the loss in *k*_cat_, because the *K*_M_ remained almost unaffected. Surprisingly, whereas Trp466His exhibited a measurable catalytic efficiency for **5b**, no activity could be found with Val465Thr, even at higher enzyme concentrations. Since for Val465Thr an overall performance similar to the wild-type HMFO was expected, it is remarkable that no activity was detected on substrate **5b**. A possible explanation is that there is an extremely low *K*_M_ and *k*_cat_, which would be in line with the observed lowered *K_M_* for the alcohol substrate with Val465Thr. It is striking that the efficiency of Trp466His for **5a** and **5b** is within a close range, between 50 s^−1^·M^−1^ and 20 s^−1^·M^−1^, respectively, but for the wild-type enzyme these values differed by three orders of magnitude.

Kinetic parameters of wild-type HMFO and variants were determined in sodium phosphate buffer (100 mM, pH 7.0) at 30 °C by monitoring H_2_O_2_ formation in a coupled assay [27].

## 3. Materials and Methods 

### 3.1. Bacterial Cultivation and Enzyme Production

Protein expression and purification were performed after an adapted protocol from Dijkman and Fraaije [4]. For HMFO expression, an overnight culture of *E. coli* BL21(DE3) cells containing the SUMO-HMFO encoding plasmid (Champion™ pET SUMO, ThermoFisher Scientific, Carlsbad, CA, USA) was diluted 1:100 in 200 mL of Terrific Broth containing 50 µg·mL^−1^ kanamycin and was grown at 37 °C until an optical density at 600 nm (OD_600_) of 0.8–1.0 was reached. Cells were induced with isopropyl-β-d-thiogalactopyranoside (IPTG, 1.0 mM) and grown overnight at 20 °C. Cells were harvested by centrifugation at 3730× *g* for 15 min (Hettich^®^ Rotina 420R centrifuge, 4 °C, Hettich, Tuttlingen, Germany) and resuspended in Tris-HCl (35 mL, 100 mM, pH 8.0) supplemented with glycerol (10% *v*/*v*), 150 mM NaCl (150 mM) and FAD (10 µM). Cells were disrupted with a Branson Digital Sonifier 250 (30% amplitude, 1 s pulse, 4 s pause for 2 min). The lysate was cleared by centrifugation (20,000× *g* for 15 min).

His-Tagged HMFO was purified by Ni-affinity chromatography (5 mL HiTrap FF column, GE Healthcare, Little Chalfont, UK) applying a 5–500 mM gradient of imidazole (binding buffer: Tris-HCl 50 mM, pH 8.0 with 150 mM NaCl containing 5 mM imidazole; elution buffer: Tris-HCl 50 mM, pH 8.0 with 150 mM NaCl containing 500 mM imidazole). Fractions containing HMFO (analysed by SDS-PAGE) were pooled, concentrated by ultrafiltration (20 mL, 50 kDa cut-off, Vivaspin, Göttingen, Germany), and desalted (Sephadex™ G-25 M, GE Healthcare, Little Chalfont, UK). The sample was lyophilized and stored at −20 °C. For activity tests, the lyophilized enzyme preparation was dissolved in sodium phosphate buffer (pH 7.0, 100 mM, 20 µL/mg) without cleaving off the SUMO-tag.

### 3.2. Site-Directed Mutagenesis

Site-directed mutagenesis of the wild-type HMFO gene was performed using two-step whole-plasmid PCR. For the creation of Val465Thr-Trp466His, the HMFO-Trp466His plasmid was used as template. The primers were ordered at IDT (Leuven, Belgium) and are listed in Table S1.

After three cycles of linear PCR, the mixture containing the forward primer and the mixture with the reverse primer were combined for additional 15 cycles. Template DNA was cleaved with *Dpn*I (New England Bio-Labs, Ipswich, MA, USA). The plasmid was purified with a PCR purification kit (Qiagen, Hilden, Germany) and transformed into *E. coli* TOP10 cells. The introduction of the mutations was confirmed by sequencing.

### 3.3. Biotransformation

Lyophilized purified HMFO variants were dissolved in phosphate buffer (100 mM, pH 7.0), after determining the protein concentration by Bradford analysis. HMFO (0.7 µM) variants and catalases from *M.* *lysodeikticus* (10 µL, 1700 U) were added to phosphate buffer (1 mL, pH 7.0, 100 mM) supplemented with FAD (20 µM). The substrate (25 mM) was added, and vials were placed into a shaker in a horizontal position. The reaction mixture was shaken at 30 °C and 120 rpm for 24 h at atmospheric oxygen concentration. The mixture was then vortexed, MeCN (500 µL) was added, the mixture was vortexed again and centrifuged, and the supernatant was directly measured by HPLC-UV.

### 3.4. Aldehyde Hydration

A solution of **1b**–**5b** (25 mM) in phosphate buffer (1 mL, 100 mM, pH 7.0) was incubated for 24 h at room temperature. After incubation, wild-type HMFO and catalase (10 µL, 1700 U) were added. The vials were placed in a shaker in a horizontal position and shaken at 30 °C and 120 rpm for 24 h at atmospheric oxygen concentration. The mixture was then vortexed, MeCN (500 µL) was added, the mixture was vortexed again and centrifuged, and the supernatant was directly measured by HPLC-UV. As a control, the reaction was performed without preincubating the aldehyde (see SI).

### 3.5. Kinetics

The *k*_cat_ and *K*_M_ values of the HMFO variants were determined by measuring H_2_O_2_ production during the reaction. In a coupled H_2_O_2_ detection assay, horseradish peroxidase (HRP 40 U/mL) oxidized 4-aminoantipyrine (0.1 mM) and 3,5-dichloro-2-hydroxybenzenesulfonic acid (1 mM) to form a pink product that was monitored at 515 nm (ε_515_ = 26 mM^−1^·cm^−1^) or with Amplex^®^ Red (50 µM, monitored at 563 nm, ε_563_ = 52 mM^−1^·cm^−1^). The activity of HMFO (0.1 to 10 µM) was assayed at different substrate concentrations (0.05 to 10 mM), depending on the substrate used. All reactions were performed at 30 °C in phosphate buffer (100 mM, pH 7.0).

## 4. Conclusions

HMFO is a potent biocatalyst for the O_2_-dependent oxidation of substituted benzylic alcohols to the corresponding aldehydes and/or carboxylic acids. The relative ratio of both oxidation steps strongly depends on the electronic properties of the *p*-substituent on the aromatic ring, which is due to its effect on the hydration of the aldehyde forming the *gem*-diol substrate for the second oxidation step. As a result, 4-nitrobenzyl alcohol was efficiently converted to the corresponding carboxylic acid. With the introduction of hydrogen bonding amino acid residues within the active site, we expected to boost the second oxidation step by improving the binding of the *gem*-diol substrate. Indeed, the relative activity of the two oxidation steps was improved up to ~5-fold in favor of over-oxidation to the carboxylic acid for substrate **5a**, albeit at the cost of overall activity. However, for substrates **1a**–**4a**, lacking an electron-withdrawing (activating) group, no such improvement was achieved due to the unfavorable position of the aldehyde-hydration equilibrium. If carboxylic acids are desired from alcohols, engineered HMFO variants showing enhanced over-oxidation activities are advantageous, because they do not form toxic levels of aldehyde intermediates through direct formation of carboxylic acids, which are better tolerated by whole-cell biocatalysts [28].

## Figures and Tables

**Table 1:** Enhanced carboxylic acid formation (**5c**) from **5a** with HMFO variants.

Variant	Ratio (%) ^a^
Wild type	6
Trp466Gln	5
Trp466His	18
Trp466Ser	18
Trp466Arg	17
Val465Ser	14
Val465Thr	7
Val465Asp	16
Val465Thr/Trp466His	37

^a^ Ratio of carboxylic acid (**5c**) to aldehyde formation (**5b**).

**Table 2:** Steady state kinetics of wild-type HMFO and selected variants in the oxidation of *p*-nitrobenzyl alcohol (**5a**) and *p*-nitrobenzaldehyde (**5b**).

Substrate		HMFO Variant	*k*_cat (*app*)_ (s^−1^)	*K*_M (*app*)_ (mM)	*k*_cat (*app*)_*/K*_M (*app*)_ (s^−1^·M^−1^)
**5a**	4-nitrobenzyl alcohol	Wild type	7.4	0.26	28,000
		Trp466His	0.02	0.40	50
		Val465Thr	1.7	0.03	57,000
**5b**	4-nitrobenzaldehyde	Wild type	0.3	0.31	970
		Trp466His	0.003	0.16	20
		Val465Thr	<0.0001	n.d.	-

## Supplementary Materials

Supplementary materials are available online.
