# Supplementary material for: Rational Engineering of a Flavoprotein Oxidase for Improved Direct Oxidation of Alcohols to Carboxylic Acids
Source: Molecules. 2017 Dec 12;22(12):2205. doi: 10.3390/molecules22122205 (PMC6149797; doi:10.3390/molecules22122205)
Supplement: Supplementary file 1 [file molecules-22-02205-s001.pdf]

Rational engineering of a flavoprotein oxidase for improved direct oxidation of alcohols to carboxylic acids

Mathias Pickl,[b] Christoph K. Winkler,[a,b] Silvia M. Glueck,[a,b] Marco W. Fraaije,[c] Kurt Faber[b*]

[a] Austrian Centre of Industrial Biotechnology, ACIB GmbH c/o

[b] Department of Chemistry, University of Graz, Heinrichstrasse 28, A-8010 Graz, Austria

[c] Molecular Enzymology Group, Groningen Biomolecular Sciences and Biotechnology Institute, University of Groningen, Nijenborgh 4, 9747 AG Groningen, The Netherlands.

Corresponding author: kurt.faber@uni-graz.at

Electronic Supplementary Material

General

Benzylic alcohols, benzaldehydes and catalase from *M. lysodeikticus* were obtained from Sigma Aldrich (Steinheim, Germany). The biotransformations were accomplished in a HT Infors Unitron AJ 260 at 120 rpm and 30 °C (vials in horizontal position). Molecular biology enzymes and stock solutions were purchased from Thermo Scientific (Vienna, Austria). All products were identified by comparison with authentic reference material.

Site-directed mutagenesis

A typical PCR mixture (25 µL) contained template DNA (10 ng), forward or reverse primer (1.25 µL, 0.1 µM) and a master mix [50 µL master mix contain Phusion Polymerase (0.5 µL), GC buffer (10 µL), DMSO (5 % v/v) and dNTPs (1 µL of 10 mM stock)]. After three cycles of linear PCR, the mixture containing the forward primer and the mixture of the reverse primer were combined. Template DNA was cleaved with *Dpn*I (New England Bio-Labs). The plasmid was purified with a PCR purification kit (Qiagen) and transformed into *E. coli* TOP10 cells. The introduction of the mutations was confirmed by sequencing (Microsynth AG, Balgach, Switzerland). The primers are listed in Table S1.

Table S1: Primers used for site-directed mutagenesis. For all primers, the mutated codon is underlined.

**Primer name**	**Sequence (from 5' to 3')**
**Trp466Phe-fw**	AACGTCGGCGGTGTTTTTCATGCGAGCGG
**Trp466Phe-rv**	CCGCTCGCATGAAAAACACCGCCGACGTT
**Trp466Ala-fw**	CGTCGGCGGTGTTGCACATGCGAGCG
**Trp466Ala-rv**	CGCTCGCATGTGCAACACCGCCGACG
**Trp466Tyr-fw**	CGTCGGCGGTGTTTATCATGCGAGCG
**Trp466Tyr-rv**	CGCTCGCATGATAAACACCGCCGACG
**Trp466Asn-fw**	CGTCGGCGGTGTTAATCATGCGAGCG
**Trp466Asn-rv**	CGCTCGCATGATTAACACCGCCGACG
**Trp466Gln-fw**	CGTCGGCGGTGTTCAACATGCGAGCG
**Trp466Gln-rv**	CGCTCGCATGTTGAACACCGCCGACG
**Trp466Ser-fw**	CGTCGGCGGTGTTAGCCATGCGAGCG
**Trp466Ser-rv**	CGCTCGCATGGCTAACACCGCCGACG
**Trp466Thr-fw**	CGTCGGCGGTGTTACCCATGCGAGCG
**Trp466Thr-rv**	CGCTCGCATGGGTAACACCGCCGACG
**Trp466Arg-fw**	CGTCGGCGGTGTTCGTCATGCGAGCG
**Trp466Arg-rv**	CGCTCGCATGACGAACACCGCCGACG
**Trp466Lys-fw**	CGTCGGCGGTGTTAAACATGCGAGCG
**Trp466Lys-rv**	CGCTCGCATGTTTAACACCGCCGACG
**Trp466Asp-fw**	CGTCGGCGGTGTTGATCATGCGAGCG
**Trp466Asp-rv**	CGCTCGCATGATCAACACCGCCGACG
**Trp466Glu-fw**	CGTCGGCGGTGTTGAACATGCGAGCG
**Trp466Glu-rv**	CGCTCGCATGTTCAACACCGCCGACG
**Trp466His-fw**	CGTCGGCGGTGTTCATCATGCGAGCG
**Trp466His-rv**	CGCTCGCATGATGAACACCGCCGACG
**Val465Ser-fw**	CGGCGGTAGCTGGCATGCGAGCGGCACG
**Val465Ser-rv**	CGTGCCGCTCGCATGCCAGCTACCGCCG
**Val465Thr-fw**	TACGAACGTCGGCGGTACCTGGCAT
**Val465Thr-rv**	ATGCCAGGTACCGCCGACGTTCGTA
**Val465Asp-fw**	TACGAACGTCGGCGGTGATTGGCAT
**Val465Asp-rv**	ATGCCAATCACCGCCGACGTTCGTA
**Val465Thr-Trp466His-fw**	GAACGTCGGCGGTACCCATCATGCG
**Val465Thr-Trp466His-rv**	CGCATGATGGGTACCGCCGACGTTC

Aldehyde hydration

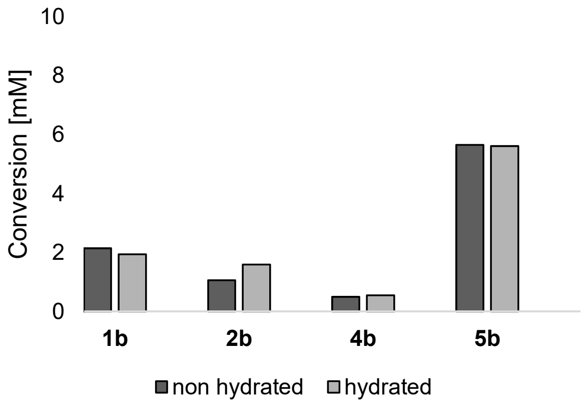

Figure S1 Conversion of benzaldehydes **1b**, **2b**, **4b** and **5b** by HMFO wild type with or without preincubation in buffer (sodium phosphate pH 7.0, 100 mM) at room temperature.

HPLC method

500 µL of the reaction mixture were diluted with 500 µl MeCN and the protein was denaturated by strong spinning of the sample. From the clear supernatant, the conversion was determined by HPLC equipped with an UV-detector, using a LUNA C18 column (Phenomenex ®) and water and MeCN containing 0.1% trifluoroacetic acid (TFA) as mobile phase with a flow rate of 1 mL/min. In the standard method 100% H_2O were used for 2 min, then a gradient from 0% MeCN to 40% MeCN over 13 min was applied, followed by a gradient to 100% MeCN within 5 min, which was kept for 2 min. Finally 100% H_2O were maintained for 3 min. The retention times are summarized in Table S2.

Table S2 Retention times of substrates and products

Compound	t_R Alcohol [min]	Aldehyde [min]	Carboxylic acid [min]
**1**	15.15	17.30	18.89
**2**	18.25	20.83	20.00
**3**	16.65	19.60	18.50
**4**	15.53	19.23	17.73
**5**	16.59	19.11	18.31

Protein–ligand docking simulations of benzaldehyde hydrate into HMFO wild type

Docking simulations were performed using the YASARA software (Version 15.3.8) with the HMFO X-ray crystal structure containing the oxidized co-factor (PDB: 4UDP) as template [1]. Substrates were docked into the active site using Autodock Vina with default settings, a cubic simulation cell of 10.0 Å around N5 of FAD and the AMBER03 force field.

Internal Plasmid Codes

Table S3 Internal Plasmid Codes

Plasmid	pEG Number
HMFO-wt	pEG387
HMFO-Trp466Ala	pEG388
HMFO-Trp466Phe	pEG389
HMFO-Trp466His	pEG390
HMFO-Trp466Tyr	pEG391
HMFO-Val465Ser	pEG392
HMFO-Val465Thr	pEG393
HMFO-Val465Thr/Trp466Phe	pEG394

Reference

1. Krieger, E.; Vriend, G.; YASARA View - molecular graphics for all devices - from smartphones to workstations. *Bioinformatics* **2014**, *30*, 2981-2982, Available online: 10.1093/bioinformatics/btu426. https://www.ncbi.nlm.nih.gov/pmc/articles/PMC4184264/ (accessed on: 20 11 2017)
